# Morphology of the tentorium in the ant genus *Lasius* Fabricius (Hymenoptera: Formicidae)

**DOI:** 10.1038/s41598-019-43175-w

**Published:** 2019-04-30

**Authors:** Hiroshi Kubota, Jin Yoshimura, Shuhei Niitsu, Akira Shimizu

**Affiliations:** 1 Department of Biological Sciences, Tokyo Metropolitan University, Hachioji, Tokyo 192-0397 Japan; 2 Graduate School of Science and Technology, and Department of Mathematical and Systems Engineering, Shizuoka University, Hamamatsu, 432-8561 Japan; 3 Department of Environmental and Forest Biology, State University of New York College of Environmental Science and Forestry, Syracuse, New York 13210 USA; 4 Marine Biosystems Research Center, Chiba University, Kamogawa, Chiba, 299-5502 Japan

**Keywords:** Taxonomy, Electron microscopy, Entomology

## Abstract

The tentorium is the internal skeleton of the head capsule of insects. Several studies have shown that the structure of the tentorium is an important factor not only for the morphology and systematics but also for the phylogeny and evolution. In ants, however, only three studies have reported tentorial morphology so far. We reveal the fundamental structure of the tentorium of the genus *Lasius* (Hymenoptera, Formicidae) and its minor variation among six species of the genus. Based on the results, we give new terminologies of the organ, presenting a schematic diagram of the tentorium. We clarify muscle attachment to the tentorium by constructing a three-dimension image of the tentorium and muscles. We then verify the attachment areas of the antennal muscles and maxillary adductor muscles on the tentorium. The results show that the muscular attachment areas are broader than previously thought. Our study indicates that the key to understanding the evolution of the tentorium is its functional morphology, in relation to the attachments of the muscles originating from the tentorium within the head capsule. This is the first report of the three-dimensional images of the ant tentorium and its attached muscles. The intra- and inter-specific variations of the tentorium is also reported for the first time.

## Introduction

In insects, the integument (body wall) is occasionally invaginated into the body cavity to form an internal, ‘skeletal’, support. Such a structure is termed an apodeme. The various apodemes together comprise the endoskeleton. The tentorium is the endoskeleton of the insect head and is composed of four parts, viz., the tentorial bridge, a pair of the anterior and posterior tentorial arms and the dorsal tentorial arms^1,2^. In addition to these, the tentorium often possesses various processes arising from the anterior tentorial arm, posterior tentorial arm or tentorial bridge in some Orthoptera^1,3,4^.

In ants, few studies have investigated the morphology of the tentorium. In his paper on the morphology of the queen of *Lasius niger* (Linnaeus), Janet^5^ presented a schematic illustration of the tentorium and gave anatomical terms to its noticeable structures. Bugnion^6^ investigated the mouthparts of a few species and presented several illustrations of the tentoria. López^7^ defined species of *Leptanilla* Emery (Leptanillinae) on the basis of several morphological features, including those of the tentorium.

Although the above studies treated the tentorium only from morphological and taxonomical viewpoints, recent entomologists have regarded it as an important organ for phylogenetic analyses and the evolution^4,8,9^. Klass and Eulitz^4^ investigated the tentoria, head sulci and anterior tentorial pits of Dictyoptera (cockroaches and mantises) and Mantophasmatodea (heel-walkers). Using characters of these structures, they analyzed the phylogenetic relationships of these orders and evaluated the homoplastic evolution of the characters. They concluded that the perforated tentorial bridge is definitely an autapomorphy of (derived feature unique to) Dictyoptera. Zimmermann *et al*.^8^ examined the neuropteran adult tentoria and muscles originating from the tentorium. Based on these tentorial characters combined with larval morphological characters, they analyzed the phylogenetic relationships of families of Neuroptera. Weide *et al*.^9^ worked out the arrangement of the tentorium and the sclerites of the hypopharynx-prementum complex in Aleocharinae (Coleoptera, Staphylinidae), presenting comparative three-dimension (3D) reconstructions of those structures. They also surmised evolutionary scenarios for some characters of those structures, together with feeding styles (spore feeding/non-spore feeding), by mapping the characters onto a phylogenetic hypothesis. Paul *et al*.^10^ investigated the functional movements of tongues in ants from the anatomical viewpoint of attached muscles. Thus the internal structures should exhibit phylogenetic and evolutionary constraints in relation to modes of life and behavior.

In the present study, we examined the tentorium of *Lasius niger*, the structure of which Janet^5^ described. He illustrated both interior and exterior outgrowths of the anterior tentorial arm (Janet^5^: pl. 5, K). In his figure, the antennal muscles originate from the interior outgrowth and the maxillary adductor muscles originate from the exterior outgrowth. Our preliminary examination suggested that the antennal muscles originate from not only the interior but also exterior outgrowths while the maxillary adductor muscles originate from the interior outgrowth. We also detected the dorsal tentorial arm that Janet^5^ did not describe.

We thus conducted the present study to: (1) present the precise morphology of the tentorium, based on workers of *Lasius niger*; (2) clarify the variation of the tentorial structure among species of the genus *Lasius* Fabricius (Table 1); (3) reveal the origins of muscles on the tentorium and the relationships between the tentorial structures and the muscle attachment. In order to understand the topology of the tentorium and the associated muscles, we construct their 3D image for *Lasius japonicas* Santschi, which is the first report of the ant tentorium. The intra- and inter-specific variation of measurements of the tentorium is also reported for the first time.

**Table 1:** Species of the genus *Lasius* examined.

	Locality	N (worker)
*Lasius* (*Dendrolasius*) *spathepus* Wheeler, 1910	Kuji, Iwate Pref., Japan	5
*Lasius* (*Chthonolasius*) sp.	Shikaoi‐cho, Hokkaido, Japan	5
*Lasius* (*Cautolasius*) *flavus* (Fabricius, 1782)	Mt. Fuji, Shizuoka Pref., Japan	5
*Lasius* (*Lasius*) *niger* (Linnaeus, 1758)	West Sussex, UK	5
*Lasius* (*Lasius*) *japonicus* Santschi, 1941	Odawara, Kanagawa Pref., Japan	5
*Lasius* (*Lasius*) *sakagamii* Yamauchi & Hayashida, 1970	Oi‐cho, Kanagawa Pref., Japan	5

All specimens are deposited at the Laboratory of Systematic Zoology, Department of Biological Sciences, Tokyo Metropolitan University, Tokyo, Japan.

## Results

### Morphology of the tentorium

#### General structure of the tentorium in the genus *Lasius*

**Figure 1:**
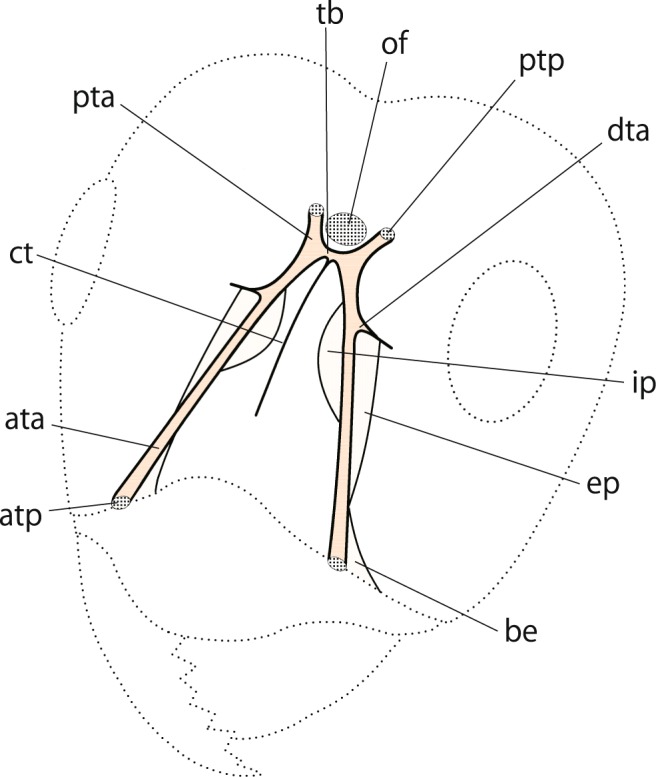
General structure of the tentorium in the genus *Lasius*. ata, anterior tentorial arm; atp, anterior tentorial pit; be, buttress-like extension; ct, corpotendon; dta, dorsal tentorial arm; ep, exterior plate; ip, interior plate; of, occipital foramen; pta, posterior tentorial arm; ptp, posterior tentorial pit; tb, tentorial bridge.

The tentorium consists of the following parts: the paired anterior tentorial arms (Fig. 1, ata), paired posterior tentorium arms (Fig. 1, pta), tentorial bridge (Fig. 1, tb), dorsal tentorial arm (Fig. 1, dta), corpotendon (Fig. 1, ct) and interior and exterior plates (Fig. 1, ip, ep). The anterior tentorial arms are tubular, invaginating from the anterior tentorial pit (Fig. 1, atp) and extend backward, a pair converging and connecting to the tentorial bridge posteriorly. The arm possesses a semicircular outgrowth interiorly, which was termed the ‘internal, roundish plates’ by López *et al*.^7^ but is here designated as the ‘interior plate’ because it is not always semicircular. The exterior outgrowth of the anterior tentorial arm, if any, is here newly termed the ‘exterior plate’. This plate is located anteriorly to the dorsal tentorial arm, narrows anteriad and twists outwardly, connecting to the buttress-like extension (Fig. 1, be). The posterior tentorial arm is also tubular, invaginating from the posterior tentorial pit (Fig. 1, ptp), a pair connecting to the tentorial bridge, and is much shorter than the anterior tentorial arm. The dorsal tentorial arm (Fig. 1, dta) is a dorsal outgrowth of the anterior tentorial arm, the apical part of which is branch-like, weakly sclerotized and does not connect to the inner wall of the cranium dorsally.

#### Comparison of the tentorial structure between species of the genus *Lasius*

**Figure 2:**
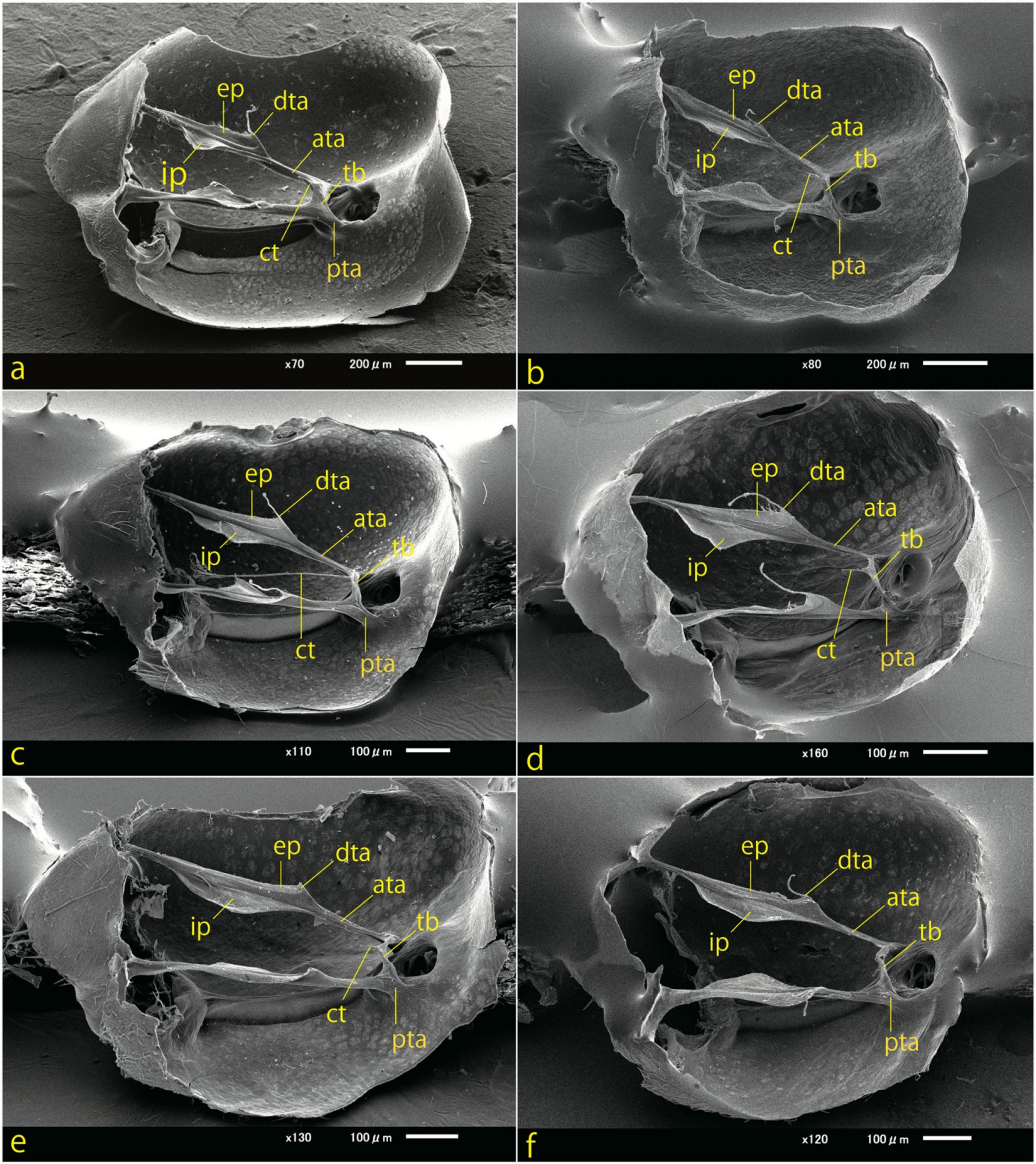
SEM images of the tentoria in the genus *Lasius*. (**a**) *L*. (*Dendrolasius*) *spathepus*. (**b**) *L*. (*Chthonolasius*) sp. (**c**) *L*. (*Cautolasius*) *flavus*. (**d**) *L*. (*Lasius*) *niger*. (**e**) *L*. (*L*.) *japonicus*. (**f**) *L*. (*L*.) *sakagamii*. ata, anterior tentorial arm; ct, corpotendon; dta, dorsal tentorial arm; ep, exterior plate; ip, interior plate; pta, posterior tentorial arm; tb, tentorial bridge. The corpotendon is wholly or partly removed in all figures except (**c**); the tip of the dorsal arm is removed in (**e**).

Scanning electron microscopy (SEM) images of the tentorium are shown in Fig. 2a–f. In all species, the tentorium is composed of the anterior tentorial arm, corpotendon, dorsal tentorial arm, exterior plate, interior plate, posterior tentorial arm and tentorial bridge. The tentoria of the six species, although structurally very similar, have minor differences, as stated below.

**Figure 3:**
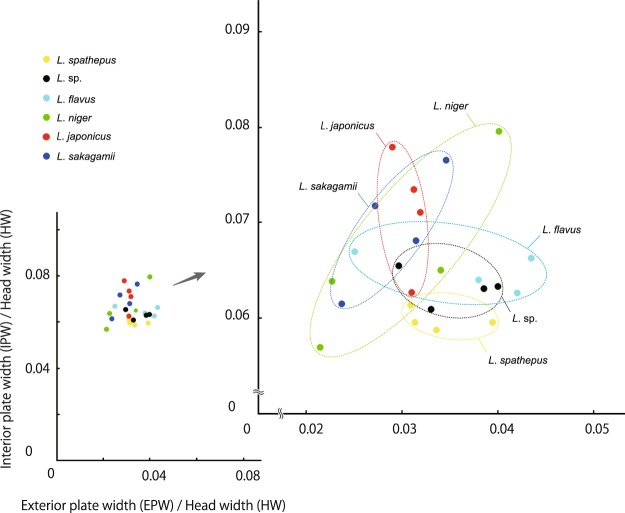
Scatter diagram, showing the ratio of interior plate width to head width against the ratio of exterior plate width to head width. Each datapoint represents one individual. Species are color coded (for original data, see Supplementary Table S1).

(a) Variation of the interior and exterior plates (Supplementary Table S1, Fig. 3, Supplementary Figs S1 and S2)

Figure 3 shows the variations of both the ratio of interior plate width to head width (IPW/HW) and the ratio of exterior plate width to head width (EPW/HW). All species overlap in their data points indicating no specific characteristics in both ratios. Surprisingly, however, it shows the following distinctive allometric relationships. In *L. niger* and *L. sakagamii* Yamauchi & Hayashida, the IPW/HW increases almost linearly against the EPW/HW. In *L. spathepus* Wheeler, *L. flavus* (Fabricius) and *L*. sp., the former is almost the same or slightly decreasing against the latter. In *L. japonicus* Santschi, the former decreases sharply against the latter; the variation in the latter is extremely small, compared with those in all other species.

**Figure 4:**
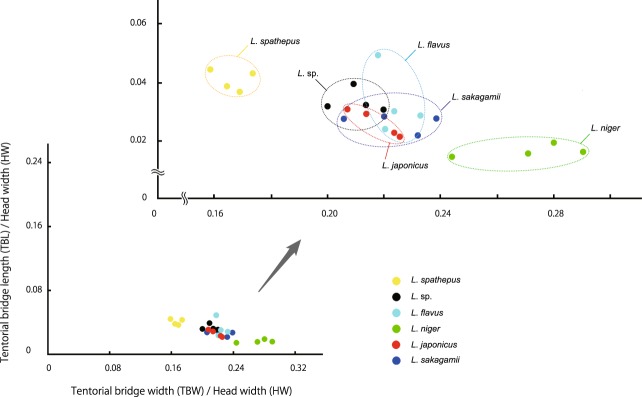
Scatter diagram, showing the ratio of tentorial bridge length to head width against the ratio of tentorial bridge width to head. Each datapoint represents one individual. Species are color coded (for original data, see Supplementary Table S1).

(b) Variation of the tentorial bridge (Supplementary Table S1, Fig. 4, Supplementary Figs S1 and S2)

The variations of both the ratio of tentorial bridge length to head width (TBL/HW) and the ratio of tentorial bridge width to head width (TBW/HW) overlap for *Lasius* sp., *L. flavus*, *L. japonicus* and *L. sakagamii*. In contrast, it is apparent that in *L. spathepus* the TBL/HW is high and the TBW/HW is very low and that in *L. niger* the former is very low and the latter is very high, compared with those in the other species. This means that the tentorial bridge is long and narrow in *L. spathepus* (Supplementary Figs S2a1–4) and short and wide in *L. niger* (Supplementary Figs S2d1–4). Moreover, in *L. niger* and *L. sakagamii*, the TBL/HW is not affected by the TBW/HW that shows a large variation. On the contrary, in *L. flavus*, the former sharply decreases against the latter that shows a small variation. In *L. japonicus*, the former gradually decreases against the latter.

#### Muscular system

**Figure 5:**
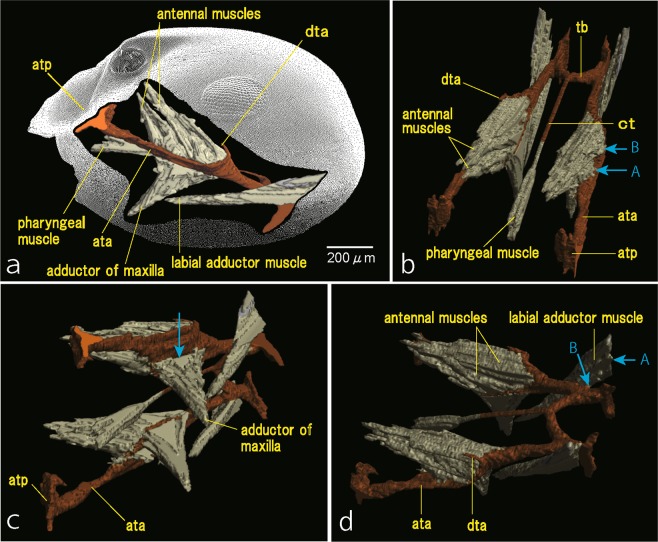
The tentorium and the attached muscles of *Lasius japonicus*. (**a**) Lateral view. (**b**) Anterodorsal view; arrow A, attachment area of anterior antennal muscles; arrow B, attachment area of posterior antennal muscles. (**c**) Anteroventral view; arrow, attachment area of maxillary adductor muscle. (**d**) Dorsolateral view; arrow A, attachment area of labial adductor muscle on the inner wall of the cranium. Janet^3^ illustrated that the labial adductor muscle originates from the posterior tentorial arm (arrow B), which was not confirmed in the present study. ata, anterior tentorial arm; atp, anterior tentorial pit; ct, corpotendon; dta, dorsal tentorial arm; tb, tentorial bridge.

Different orientations of the tentorium and muscles are shown in Fig. 5a–d. The antennal muscles are divided into two groups (Fig. 5a): one is the anterior muscles originating from the dorsal faces of the interior and exterior plates anteriorly (Fig. 5b, arrow A), which are assumedly equivalent to the ‘depressor muscles’ *sensu* Deshpande^11^ and ‘levator and protractor’ *sensu* Ehmer & Gronenberg^12^; the other is the posterior muscles originating from the dorsal faces of both plates posteriorly (Fig. 5b, arrow B), which correspond to ‘levator muscles’^11^ and ‘depressor and retractor’^12^. The use of the levator, protractor, depressor and retractor indicates the function of muscles; we here only refer these muscles based on the positions (anterior or posterior), because we cannot evaluate the functions of these muscles directly.

In contrast, a group of the maxillary adductor muscles originate from the ventral face of the interior plate (Fig. 5c, arrow). The labial adductor muscle originates from the inner wall of the cranium immediately outside the posterior arm (Fig. 5d, arrow A) and a small amount of the pharyngeal muscle attaches to the tip of the corpotendon (Fig. 5b).

## Discussion

### Variation of the tentorial structure

In all species of the genus *Lasius* examined, the tentorium is composed of the anterior and posterior tentorial arms, interior and exterior plates, tentorial bridge, dorsal tentorial arms and corpotendon. In their figures, López *et al*.^7^ illustrated that six species of the ant genus *Leptanilla* (Leptanillinae) have the anterior and posterior tentorial arms, interior plate (‘internal roundish plate’), tentorial bridge and dorsal tentorial arm (‘tooth-like protuberance’) in common but did not show the exterior plate in any species. It is thus found that the latter plate is lacking in this genus. These suggest that the composition of the tentorium is stable in closely related species, such as congeneric species. On the contrary, the composition of the tentorium may differ among higher taxa because the exterior plate, for example, is developed in *Lasius* but is lacking in *Leptanilla*. According to recent molecular phylogenetic analyses of ants, *Leptanilla* forms one of the most basal clades, while *Lasius* is placed in one of the most distal clades^13,14^. Based on these hypotheses, in the next stages, we will be able to infer the evolutionary processes of the tentorial morphological features.

López *et al*.^7^ found morphological differences in the tentorium among species of *Leptanilla*. *Leptanilla theryi* Forel differs from *L. plutonia* López and *L. ortunoi* López in its tooth-like, thin and pedunculate dorsal tentorial arm. *Leptanilla nana* Santschi differs from *L. zaballosi* Barandica and *L. charonea* Barandica in the dorsal tentorial arm not tooth-like but small and sharply pointed and the interior plate not roundish but extended anteriorly. They used these differences in species diagnoses.

Our study revealed that the shapes of the tentorium might be useful traits from the viewpoint of functional morphology. For example, the sample distributions of the IPM/HW against the EPW /HW exhibit positive correlations in *L. niger* and *L. sakagamii*, while almost no correlation (horizontal distributions) in *L. flavus*, *L. spathepus* and *L*. sp., and a vertical distribution in *L. japonicus* (Fig. 3). Similarly, the sample ranges of the TBL/HW against the TBW/HW in *L. niger* and *L. spathepus* does not overlap with those in the other species of *Lasius* (Fig. 4). These different allometric correlations in these scattered diagrams (e.g., linear increasing, linear decreasing, vertical or drastic decreasing and horizontal), once confirmed by many samples, may represent some unknown functional differences among species (Figs 3 and 4). With much more samples, in the future, we will be able to verify these unique allometric relationships for each species. The current findings may imply that the allometric relationships of morphological traits among individual specimens provide a new useful method to study the systematics and functional morphology of Formicidae.

### Muscle attachment and morphology of the tentorium

**Figure 6:**
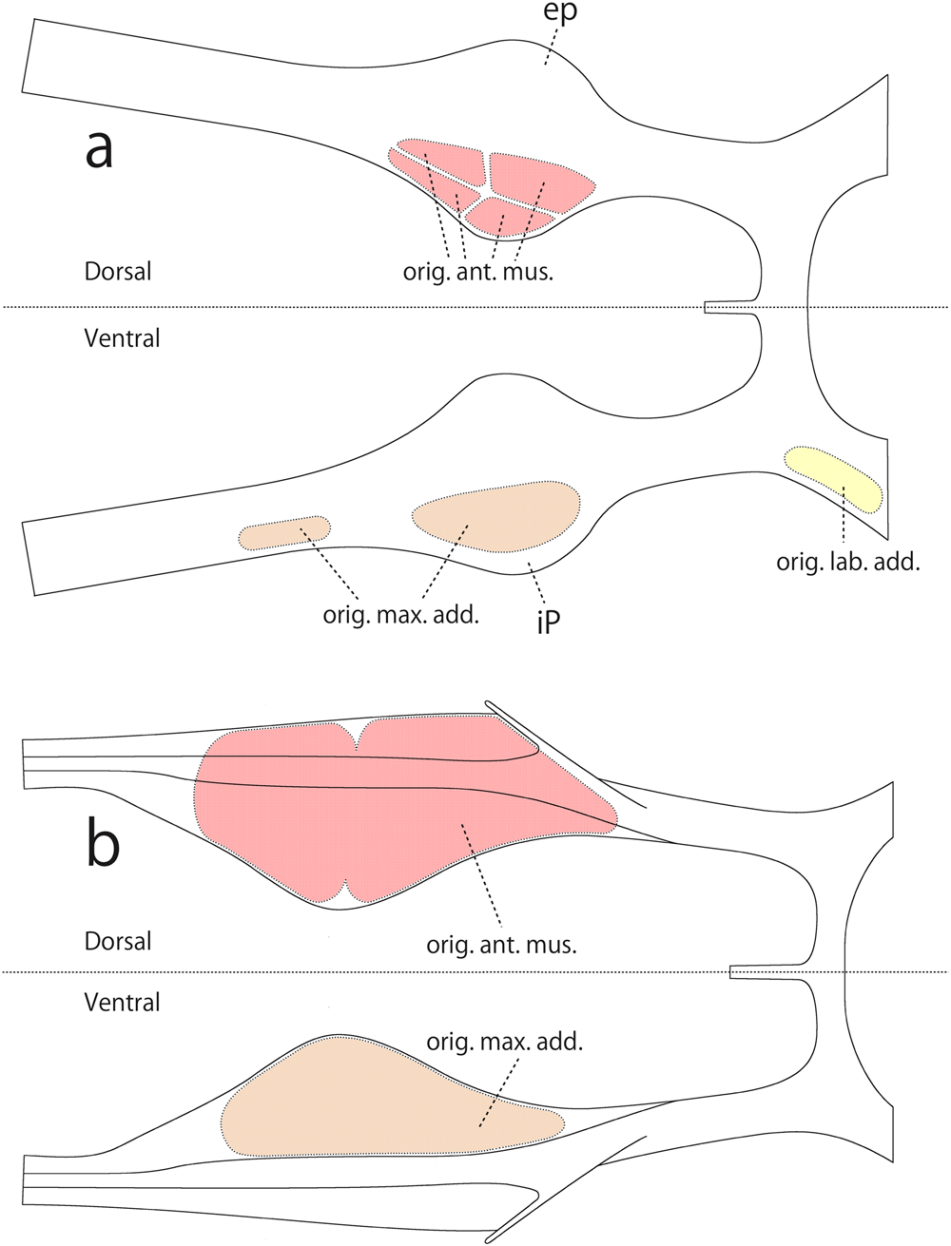
Schematic diagram of the tentorium and the origins of muscles. (**a**) Redrawn from Janet^3^. (**b**) Results of the present study. ep, Exterior plate; ip, interior plate; (orig. ant. mus.), the origins of the antennal muscles; (orig. lab. add.), the origins of the labial adductor muscles; (orig. max. add.), the origins of the maxillary adductor muscles.

Janet^5^ illustrated that several muscles originate from the tentorium of *Lasius niger*, i.e., the antennal muscles from the dorsal face of the interior plate (‘interior outgrowth’) (Fig. 6a, orig. ant. mus.), maxillary adductor muscles from the ventral face of the exterior plate (‘exterior outgrowth’) (Fig. 6a, orgi. max. add.) and adductor muscle of the labium from the ventral face of the posterior anterior arm (Fig. 6a, orig. lab. add.). However, in the present study on *L. japonicus*, we found that the antennal muscles originate from almost the entire dorsal faces of the interior and exterior plates (Fig. 6b, orig. ant. mus.), the maxillary adductor muscles originate from the ventral face of the interior plate (Fig. 6b, orig. max. add.) and the labial adductor muscle originates from the inner wall of the cranium immediately outside the posterior arm (Fig. 5d, arrow A).

As stated above, we confirmed that the tentorial structure of *L. japonicus* is almost the same as those of the other species of the genus, including *L. niger*, with minor variations. This indicates that the attachment areas of the antennal muscles and maxillary adductor muscles on the tentorium are identical in the genus. Janet’s^5^ recognition of these muscle attachments needs revising.

In *Leptanilla* the exterior plate is lacking unlike in *Lasius*. It is certain that the attachment points of the muscles homologous with the muscles originating from the exterior plate (part of antennal muscles) in *Lasius* have shifted to another part of the tentorium. According to Matsuda^2^, the muscles have often shifted their points of origin or insertion or both, accompanied by displacement of the structures to which the muscles were originally attached. The differences in the structure of the tentoria of *Lasius* and *Leptanilla* suggest that such a shift may have occurred in the antennal muscles. Furthermore, the change of the attachment points of the antennal muscles may be caused by the space-arrangement interaction with other tissues, such as the muscles of the mandible (occupying a large space inside the head), pharynx, esophagus or nervous system.

Studies on the tentorium in ants have hitherto focused on the description of its anatomical structure in relation to systematics. Our study, however, suggests that the key to understanding the evolution of the tentorium is its functional morphology, in relation to the attachments of the various muscles of the head and the spatial relationships of the various organs within the head capsule. As part of ongoing work, we will treat other morphological types of tentorium and discuss the evolution of the ant tentorium in relation to muscle attachment and other organs from the viewpoint of functional morphology.

Our analyses suggest that internal morphology is equally important compared with external morphology to understand their functional relationships and evolution. Due to the difficulty of examining the internal morphology of insects, we have extremely limited literature for the last half century. By the recent advancement of microscope technology, the examination of internal structure has become possible as Paul *et al*.^10^ studied the functional morphology of the tongue muscles in ants. Our studies suggest that the muscle attachment on the tentorium shifts among higher taxa. In the future, after examining both internal and external structures of the same specimens, we may be able to explain the functional mechanisms of the internal morphology. Thus, the study of Paul *et al*.^10^ and ours indicate new possible directions in morphological studies of insects and invertebrates.

## Materials and Methods

The Formicidae comprise about 12,000 extant, described, species belonging to 16 subfamilies, worldwide^15^. The genus *Lasius* belongs to the tribe Lasiini of the subfamily Formicinae. This tribe contains 10 genera, and *Lasius* is composed of four subgenera i.e., *Dendrolasius* Ruzsky, *Chthonolasius* Ruzsky, *Cautolasius* Wilson, *Austrolasius* Faber and *Lasius*^16^, containing 86 extant species^17^. As material, we chose six species belonging to four subgenera of the genus *Lasius*, including *L*. (*Lasius*) *niger* (Table 1). Specimens of *L. niger* were collected from England and those of the other species from Japan. All of them were adult workers.

For SEM imaging, the head was cut off from a specimen preserved in 80% ethanol, and then the mouthparts and antennae were removed from the head. The muscles of the head were (1) dissolved in 20% KOH for about three days; (2) washed with water for an hour; (3) dehydrated by using a graded series of ethanol: 80, 90, 100% with a day each; (4) immersed in t-Butanol for a day and repeated this operation once more (all of the above operations were performed at the room temperature of about 20 °C); and (5) dried with a JFD-300(JEOL) freeze dryer. Then, the head was fixed to a specimen stub with the ventral face beneath, and its right half was embedded in epoxy resin. After the resin was cured, the head was dissected under a stereoscopic microscope (Olympus SZ60). The corpotendon (a sclerotized tendon elongated anteriorly from the tentorial bridge; Fig. 1, ct) was removed from the head, except for in *L. flavus* (Fig. 2c). The tentorium was coated with platinum by using a sputter coater (JEOL JFC-1600), observed using a SEM (JEOL JSM-6510LV) and photographed dorsolaterally.

A 3D image of the tentorium and its musculature of *L. japonicus* was constructed to reveal the placement and attachment of muscles originating from the tentorium. The head was detached from the specimen preserved in 80% ethanol, immersed in 30% hydrogen peroxide for a week to soften its integument. By this treatment, the integument was decolorized. Next, dehydration was conducted with a series of ethanol: 80, 90, 100% with an hour each. The head was then immersed in 100% propylene oxide for a day. After the repletion repetition of this operation once more, the material was immersed in a solution of Epon 812 (TAAB Co., Ltd): propylene oxide = 1: 1 for two days, and then immersed in 100% Epon 812 for five days. Finally, it was embedded in a silicone mold filled with Epon 812 and was placed in an incubator set at 65 °C overnight to cure the resin. All operations were done at room temperature (about 15 °C), except for the final resin curing procedure. Since the insect cuticle is hard to section without deforming and tearing, we made relatively thick section (10 µm) using an ultra-microscope (Leica UC7).

A series of sections were mounted on microscopic slides by heat fixing (90 °C for 10 seconds), stained with Azur-B and washed in running water for a few seconds. After drying, the preparations were photographed with a digital camera (Nikon COOLPIX S5200) equipped for a transmitted light microscope (Nikon ECLIPSE E100). Using image processing software Hanako ver. 1, picture areas other than those of the tentorium and the attached muscles were deleted and color conversion was performed for each image. Since 23 out of 47 sections on one side were clear, these were also used in reverse order as substitutes for the 23 images of the opposite side. Next, a 3D image was constructed using the stereoscopic construction software Delta Viewer, based on a total of 47 images. Because these were very thin and unclear, the gastrointestinal tract muscles were excluded from our analyses. The observed head on the stub was kept in an auto drying cabinet and the whole specimen except the head was preserved in 80% ethanol.

To examine the intra- and inter-specific variations of the tentorium, preparations were made as follows: We (1) boiled the materials in 20% KOH for a few minutes; (2) washed them with water for 10 minutes; (3) extracted the tentorium from them; and (4) mounted it in gum-chloral (chloral hydrate) on a slide glass. The images were obtained with a Sanato’s USB Digital Eyepiece Camera (MEE-500B) equipped with a transmitted light microscope (Olympus BX51), and the following measurements were taken with the software Micro Capture: exterior plate width, interior plate width, tentorial bridge length, tentorial bridge width (Supplementary Fig. S1) and head width.

## Supplementary information

Supplementary Information

## Data Availability

All relevant data are within the paper.

## References

[1] Snodgrass, R. E. *Principles of Insect Morphology* (McGraw-Hill, New York 1935).

[2] Matsuda R (1965). Morphology and evolution of the insect head. Memoirs of the American Entomological Institute.

[3] Hudson GB (1945). A study of the tentorium in some orthopteroid Hexapoda. Journal of the Entomological Society of Southern Africa.

[4] Klass D, Eulitz U (2007). The tentorium and anterior head sulci in Dictyoptera and Mantophasmatodea (Insecta). Zoologischer Anzeiger.

[5] Janet, C. *Anatomie de la T****ê****te du Lasius niger* (Ducourtieux et Gout, Limoges 1905).

[6] Bugnion E (1930). Les pièces buccales, le sac infrabuccal et le pharynx des fourmis. *Bulletin*. Société Entomologique d’Egypte.

[7] López F, Martínez MD, Barandica JM (1994). Four new species of the genus *Leptanilla* (Hymenoptera: Formicidae) from Spain – relationships to other species and ecological issues. Sociobiology.

[8] Zimmermann D, Randolf S, Metscher BD, Aspöck U (2011). The function and phylogenetic implications of the tentorium in adult Neuroptera (Insecta). Arthropod Structure and Development.

[9] Weid D, Thayer MK, Betz O (2014). Comparative morphology of the tentorium and hypopharyngeal-premental sclerites in sporophagous and non-sporophagous adult Aleocharinae (Coleoptera: Stapylinidae). Acta Zoologica.

[10] Paul J, Roces F, Hölldobler B (2002). How do ants stick out their tongues?. Journal of Morphology.

[11] Deshpande SB (1984). Studies on the morphological evolutionary trends of antennary muscular patterns in insects. Journal of Animal Morphology and Physiology.

[12] Ehmer B, Gronenberg W (1997). Antennal muscles and fast antennal movements in ants. Journal of Comparative Physiology B.

[13] Moreau CS, Bell CD, Vila R, Archibald SB, Pierce NP (2006). Phylogeny of the ants: Diversification in the age of angiosperms. Science.

[14] Borowiec ML (2019). Compositional heterogeneity and outgroup choice influence the internal phylogeny of the ants. Molecular Phylogenetics and Evolution.

[15] Bolton B (2003). Synopsis and classification of Formicidae. Memoirs of the American Entomological Institute.

[16] Wilson EO (1955). A monographic revision of the ant genus *Lasius*. *Bulletin of the Museum of Comparative*. Zoology.

[17] Janda M, Folková D, Zrzavý J (2004). Phylogeny of *Lasius* ants based on mitochondrial DNA and morphology, and the evolution of social parasitism in the Lasiini (Hymenoptera: Formicidae). Molecular Phylogenetics and Evolution.

