# Supplementary material for: Morphology of the tentorium in the ant genus Lasius Fabricius (Hymenoptera: Formicidae)
Source: Sci Rep. 2019 Apr 30;9:6722. doi: 10.1038/s41598-019-43175-w (PMC6491587; doi:10.1038/s41598-019-43175-w)
Supplement: Supplementary file 1 — Supplementary Information [file 41598_2019_43175_MOESM1_ESM.pdf]

# Morphology of the tentorium in the ant genus *Lasius* Fabricius (Hymenoptera: Formicidae)

Hiroshi Kubota, Jin Yoshimura, Shuhei Niitsu and Akira Shimizu

## Supplementary Information

Supplementary Table S1. Measurements of exterior plate with (EPW), interior plate width (IPW), tentorial bridge length (TBL), tentorial bridge width (TBW) and head width (HW), and ratios of EPW/HW, IPW/HW , TBL/HW and TBW/HW. For the measurement sites, see Supplementary Fig. S1; measurement was based on materials shown in Supplementary Fig. S2.

| specise | No. Individual | EPW (㎛) | | IPW (㎛) | | TBL (㎛) | | TBW (㎛) | HW (㎛) | EPW(avg.) / HW | IPW(avg.) / HW | TBL(avg.) / HW | TBW / HW |
|---|---|---|---|---|---|---|---|---|---|---|---|---|---|
| | | Right | Left | Righ | Left | Right | Left | | | | | | |
| *L. spathepus* | a1 | 43.4 | 47.5 | 80.6 | 78.4 | 60.1 | 56.0 | 235 | 1353 | 0.034 | 0.059 | 0.043 | 0.17 |
| | a2 | 44.0 | 40.7 | 86.2 | 81.7 | 49.0 | 57.1 | 225 | 1369 | 0.031 | 0.061 | 0.039 | 0.16 |
| | a3 | 54.6 | 54.7 | 78.2 | 86.9 | 63.1 | 60.3 | 220 | 1386 | 0.039 | 0.060 | 0.045 | 0.16 |
| | a4 | 48.3 | 40.6 | 86.2 | 82.9 | 53.4 | 50.5 | 240 | 1420 | 0.031 | 0.060 | 0.037 | 0.17 |
| *L.* sp. | b1 | 28.6 | 34.8 | 70.6 | 69.5 | 33.3 | 32.3 | 235 | 1069 | 0.030 | 0.066 | 0.031 | 0.22 |
| | b2 | 34.0 | 38.9 | 68.5 | 65.8 | 44.2 | 43.3 | 231 | 1102 | 0.033 | 0.061 | 0.040 | 0.21 |
| | b3 | 45.5 | 44.0 | 71.9 | 69.8 | 36.5 | 34.7 | 224 | 1119 | 0.040 | 0.063 | 0.032 | 0.20 |
| | b4 | 44.0 | n. a. | 69.1 | 75.4 | 33.5 | 39.7 | 245 | 1144 | 0.038 | 0.063 | 0.032 | 0.21 |
| *L. flavus* | c1 | 29.5 | 34.3 | 56.5 | 38.7 | 20.7 | 22.8 | 177 | 760 | 0.042 | 0.063 | 0.029 | 0.23 |
| | c2 | 29.2 | 33.6 | 57.4 | 48.5 | 22.1 | 27.8 | 185 | 827 | 0.038 | 0.064 | 0.030 | 0.22 |
| | c3 | 22.4 | 20.1 | 57.7 | 56.0 | 41.7 | 42.1 | 185 | 848 | 0.025 | 0.067 | 0.049 | 0.22 |
| | c4 | 37.0 | 37.0 | 59.1 | 53.8 | 21.1 | 20.1 | 188 | 852 | 0.043 | 0.066 | 0.024 | 0.22 |
| *L. niger* | d1 | 24.5 | 23.6 | 48.3 | 47.4 | 10.7 | 8.8 | 175 | 601 | 0.040 | 0.080 | 0.016 | 0.29 |
| | d2 | 14.0 | 13.4 | 39.0 | 37.9 | 9.2 | 10.0 | 163 | 603 | 0.023 | 0.064 | 0.016 | 0.27 |
| | d3 | 22.6 | 19.4 | 37.5 | 42.9 | 12.3 | 11.9 | 173 | 618 | 0.034 | 0.065 | 0.020 | 0.28 |
| | d4 | 14.1 | 14.1 | 39.8 | 34.9 | 9.0 | 10.2 | 160 | 656 | 0.022 | 0.057 | 0.015 | 0.24 |
| *L. japonicus* | e1 | 21.4 | 27.7 | 63.4 | 68.6 | 16.8 | 20.1 | 191 | 847 | 0.029 | 0.078 | 0.022 | 0.23 |
| | e2 | 31.1 | 27.1 | 55.8 | 62.2 | 21.5 | 21.6 | 210 | 940 | 0.031 | 0.063 | 0.023 | 0.22 |
| | e3 | 34.2 | 29.5 | 76.5 | 73.5 | 32.8 | 30.6 | 211 | 1020 | 0.031 | 0.074 | 0.031 | 0.21 |
| | e4 | 31.0 | 34.7 | 77.3 | 69.5 | 30.8 | 29.8 | 220 | 1032 | 0.032 | 0.071 | 0.029 | 0.21 |
| *L. sakagamii* | f1 | 24.2 | 26.7 | 68.7 | 65.3 | 26.8 | 25.1 | 223 | 934 | 0.027 | 0.072 | 0.028 | 0.24 |
| | f2 | 30.5 | 30.7 | 67.4 | 65.1 | 21.5 | 21.8 | 226 | 974 | 0.031 | 0.068 | 0.022 | 0.23 |
| | f3 | 35.2 | n. a. | 78.2 | 77.2 | 26.3 | 29.9 | 209 | 1015 | 0.035 | 0.077 | 0.028 | 0.21 |
| | f4 | 26.4 | 22.6 | 64.3 | 62.9 | 29.5 | 29.6 | 227 | 1034 | 0.024 | 0.062 | 0.029 | 0.22 |

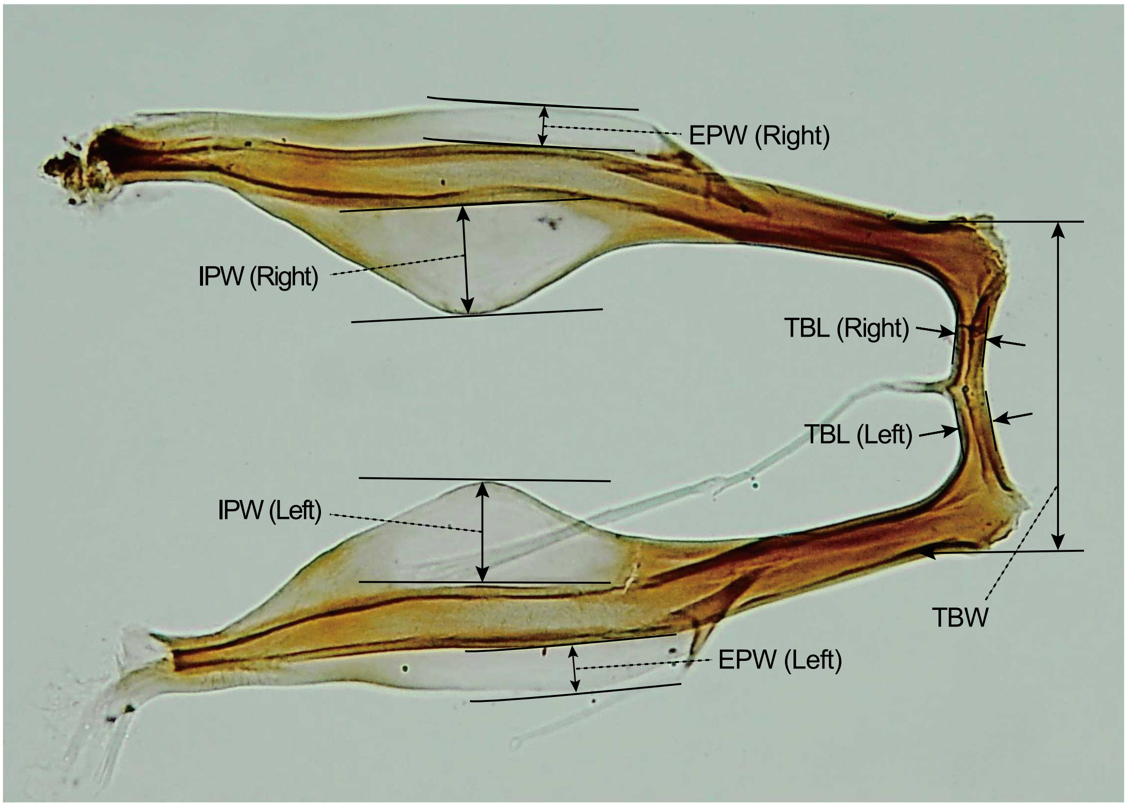


Supplementary Fig. S1. Measurements of the tentorium; EPW, Exterior plate width. IPW, Interior plate width. TBL, Tentorial bridge length. TBW, Tentorial bridge width.

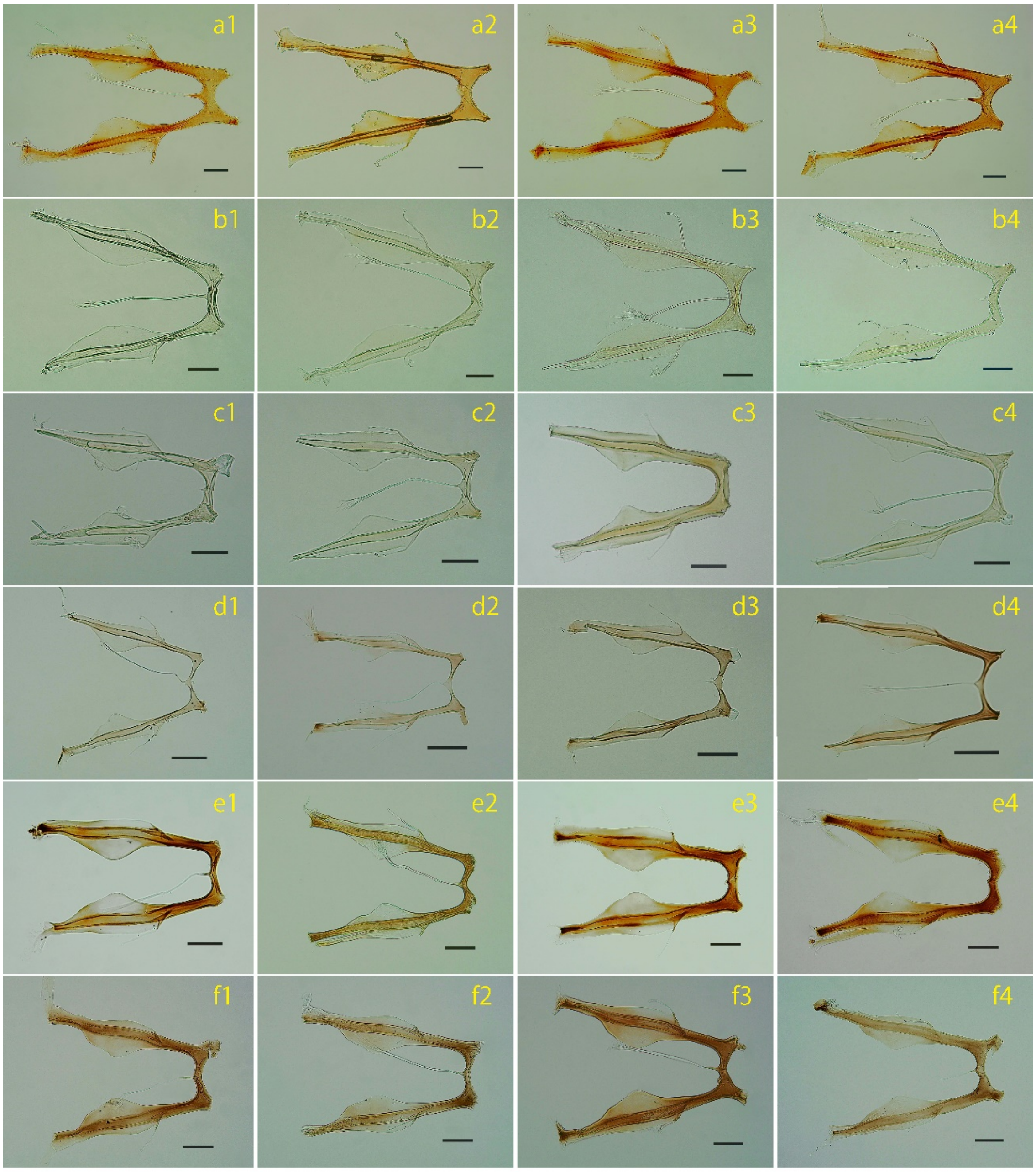


Supplementary Fig. S2. Tentoria on prepared microscope slides used for measurement. a1–4, *Lasius spathepus*. b1–4, *L.* sp. c1–4, *L. flavus*. d1–4, *L. niger*. e1–4, *L. japonicus*. f1–4, *L. sakagamii*. Codes consisting of alphabets and numerals represent individuals in Table S1. Scales: 100μm.
